# Supplementary material for: TOFIMS mass spectrometry-based immunopeptidomics refines tumor antigen identification
Source: Nat Commun. 2023 Nov 17;14:7472. doi: 10.1038/s41467-023-42692-7 (PMC10656517; doi:10.1038/s41467-023-42692-7)
Supplement: Supplementary file 1 — Supplementary Information [file 41467_2023_42692_MOESM1_ESM.pdf]

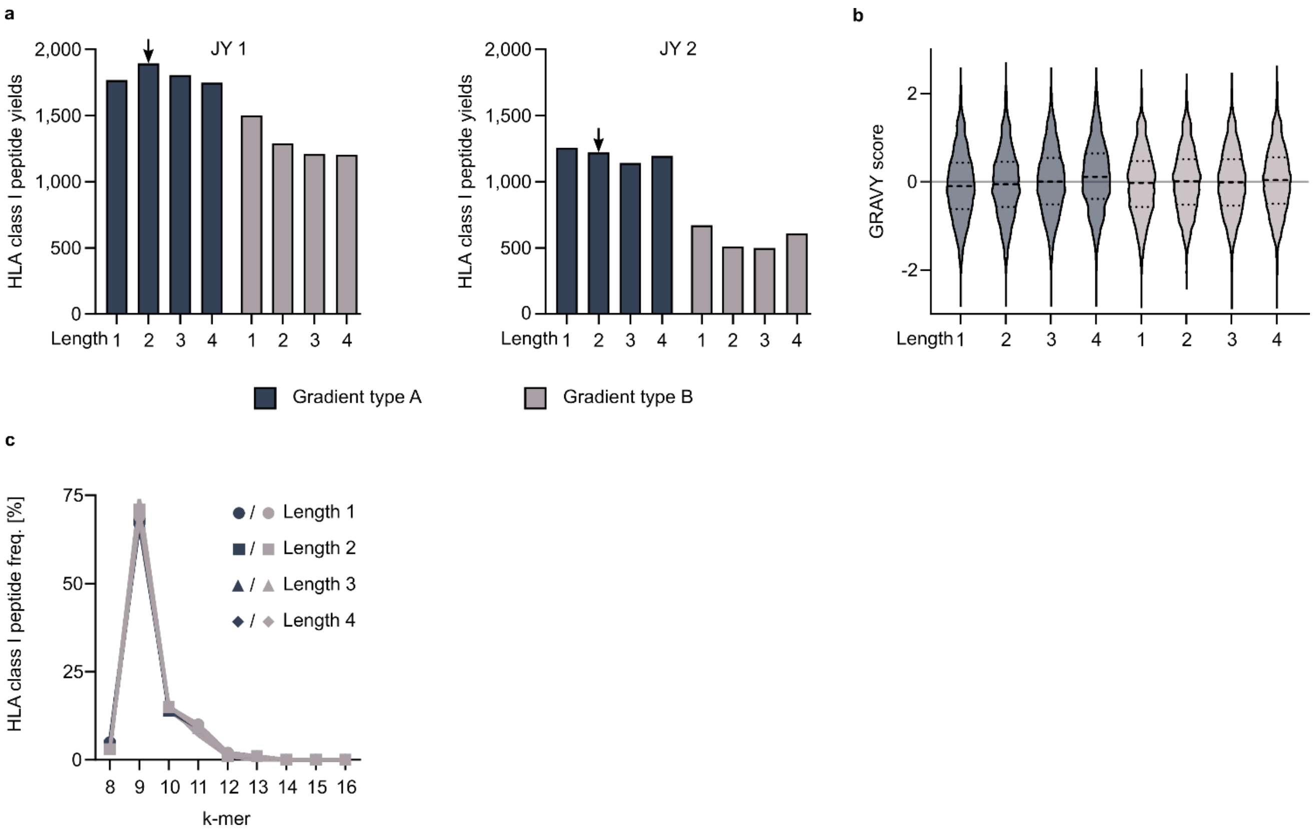

Supplementary Fig. 1 | Method development for immunopeptidomics using liquid chromatography coupled TOF_{IMS} MS. a, HLA class I peptide yields according to different gradient lengths (1, 2, 3, and 4) and gradient types (A, B) for different JY cell line sample concentrations, JY 1 (left) and JY 2 (right). **b,** Violin plots of grand average of hydrophobicity (GRAVY) score of HLA class I-presented peptides identified using different gradient lengths (1, 2, 3, and 4) and gradient types (A, B) from JY 2 sample. Median and 25th to 75th percentiles are indicated by dashed and dotted lines, respectively. **c,** Length distribution of identified HLA class I-presented peptides using the different gradient lengths (1, 2, 3 and 4) and types (A and B). Abbreviations: HLA, Human leukocyte antigen; GRAVY, grand average of hydropathy; freq., frequency. Source data are provided as a Source Data file.

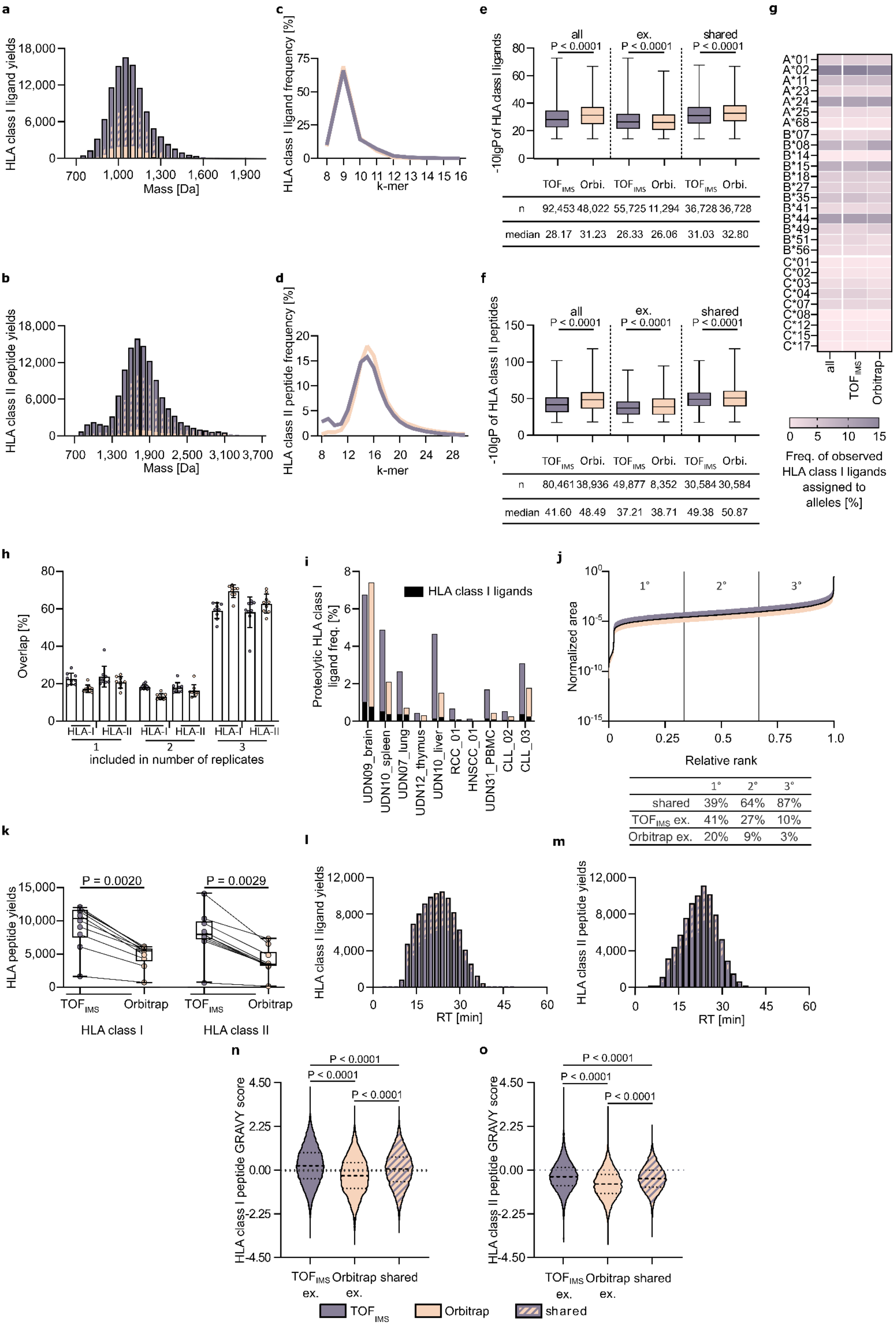
a
HLA class I ligand yields
Mass [Da]
b
HLA class II peptide yields
Mass [Da]
c
HLA class I ligand frequency [%]
k-mer
d
HLA class II peptide frequency [%]
k-mer
e
-10lgP of HLA class I ligands
all P < 0.0001
ex. P < 0.0001
shared P < 0.0001
TOF_IMS Orbi. TOF_IMS Orbi. TOF_IMS Orbi.
n 92,453 48,022 55,725 11,294 36,728 36,728
median 28.17 31.23 26.33 26.06 31.03 32.80
f
-10lgP of HLA class II peptides
all P < 0.0001
ex. P < 0.0001
shared P < 0.0001
TOF_IMS Orbi. TOF_IMS Orbi. TOF_IMS Orbi.
n 80,461 38,936 49,877 8,352 30,584 30,584
median 41.60 48.49 37.21 38.71 49.38 50.87
g
A*01 A*02 A*11 A*23 A*24 A*25 A*68 B*07 B*08 B*14 B*15 B*18 B*27 B*35 B*41 B*44 B*49 B*51 B*56 C*01 C*02 C*03 C*04 C*07 C*08 C*12 C*15 C*17
all TOF_IMS Orbitrap
Freq. of observed HLA class I ligands assigned to alleles [%]
h
Overlap [%]
HLA-I HLA-II HLA-I HLA-II HLA-I HLA-II
included in number of replicates
i
Proteolytic HLA class I ligand freq. [%]
HLA class I ligands
UDN09_brain UDN10_spleen UDN07_lung UDN12_thymus UDN10_liver RCC_01 HNSCC_01 UDN31_PBMC CLL_02 CLL_03
j
Normalized area
1° 2° 3°
Relative rank
1° 2° 3°
shared 39% 64% 87%
TOF_IMS ex. 41% 27% 10%
Orbitrap ex. 20% 9% 3%
k
HLA peptide yields
P = 0.0020
P = 0.0029
TOF_IMS Orbitrap TOF_IMS Orbitrap
HLA class I HLA class II
l
HLA class I ligand yields
RT [min]
m
HLA class II peptide yields
RT [min]
n
HLA class I peptide GRAVY score
P < 0.0001
TOF_IMS ex. Orbitrap ex. shared
o
HLA class II peptide GRAVY score
P < 0.0001
TOF_IMS ex. Orbitrap ex. shared
TOF_IMS Orbitrap shared

Supplementary Fig. 2 | Characterization of peptides identified by TOF$_{IMS}$. a, b, Mass distribution of exclusive TOF$_{IMS}$, Orbitrap or shared HLA class I (e) and II peptides (f) across all samples (n=10). **c, d,** Length distribution of TOF$_{IMS}$- or Orbitrap-identified HLA class I (c) and II peptides (d) of all analyzed samples (n=10). **e, f,** -10lgP of TOF$_{IMS}$ and Orbitrap HLA class I (c) and II peptides (d) for total (all), exclusive (ex.) and shared peptides, respectively across samples (n=10). Boxes represent median and 25th to 75th percentiles, whiskers are minimum to maximum, unpaired Kruskal-Wallis t-test. **g,** Heatmap of HLA allotype distribution across HLA class I ligands from all samples (n=10) identified by TOF$_{IMS}$ and Orbitrap. **h,** Median overlap percentage (±standard deviation) between technical triplicates per sample of HLA class I and II peptides acquired using TOF$_{IMS}$ and Orbitrap. **i,** Frequency of proteolytic HLA class I peptides per sample identified by TOF$_{IMS}$ and Orbitrap. Black indicates the percentage of HLA class I-presented peptides annotated to the respective HLA allele (ligands). **j,** Ranked normalized area of TOF$_{IMS}$ exclusive (top), Orbitrap exclusive (bottom) and shared (line) HLA class I ligands identified primary samples (n=10). The relative rank is separated into thirds (vertical lines). Percentages of ligands included in the 1st, 2nd and 3rd third relative rank are indicated in the table. **k,** HLA class I and II peptide yields identified in samples (n=10) using TOF$_{IMS}$ and Orbitrap MS. Boxes represent median and 25th to 75th percentiles, whiskers minimum to maximum, paired Kruskal-Wallis t-test. **l, m,** Number of unique HLA class I (l) and II peptide (m) identifications by TOF$_{IMS}$ in all samples (n=10) across the chromatographic gradient. **n, o,** GRAVY score of exclusive TOF$_{IMS}$, Orbitrap and shared unique HLA class I (e) and II peptides (f) across samples (n=10). Violin plots with median, 25th to 75th percentiles and unpaired Kruskal-Wallis t-test. Abbreviations: RT, retention time; Da, Dalton; norm, normalized; RCC; renal cell carcinoma; HNSCC, head and neck squamous-cell carcinoma; PBMC, peripheral blood mononuclear cell; CLL, chronic lymphocytic leukemia; GRAVY, grand average of hydrophobicity. Source data are provided as a Source Data file.

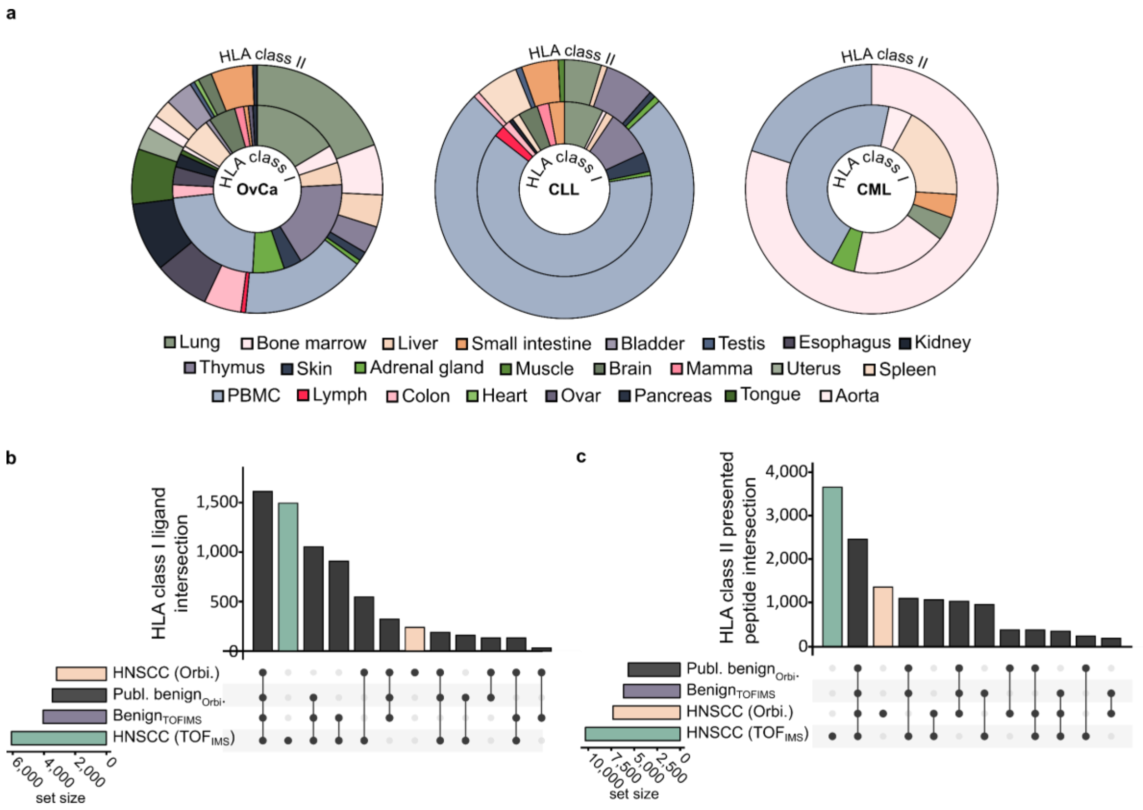

Supplementary Fig. 3 | TOF$_{IMS}$ technology enables increased tumor-associated HLA class I and HLA class II-presented peptide identifications. a, pie chart of tissue origin of rejected tumor-associated antigens of the comparative immunopeptidome profiling of published HLA class I (inner circle) and HLA class II (outer circle) for OvCa, CLL and CML. **b, c,** UpSet plots showing HLA class I ligand (b) and HLA

class II peptide (c) intersection size between peptides identified from one head and neck squamous cell carcinoma (HNSCC) primary sample using TOF_{IMS} and Orbitrap mass spectrometers and their overlap with $benign_{TOFIMS}$ dataset and published benign Orbitrap dataset. Abbreviations: HLA, Human leukocyte antigen; OvCa, ovarian carcinoma; CLL, chronic lymphocytic leukemia; CML, chronic myeloid leukemia; HNSCC, head and neck squamous cell carcinoma; publ, published; Orbi, Orbitrap. Source data are provided as a Source Data file.

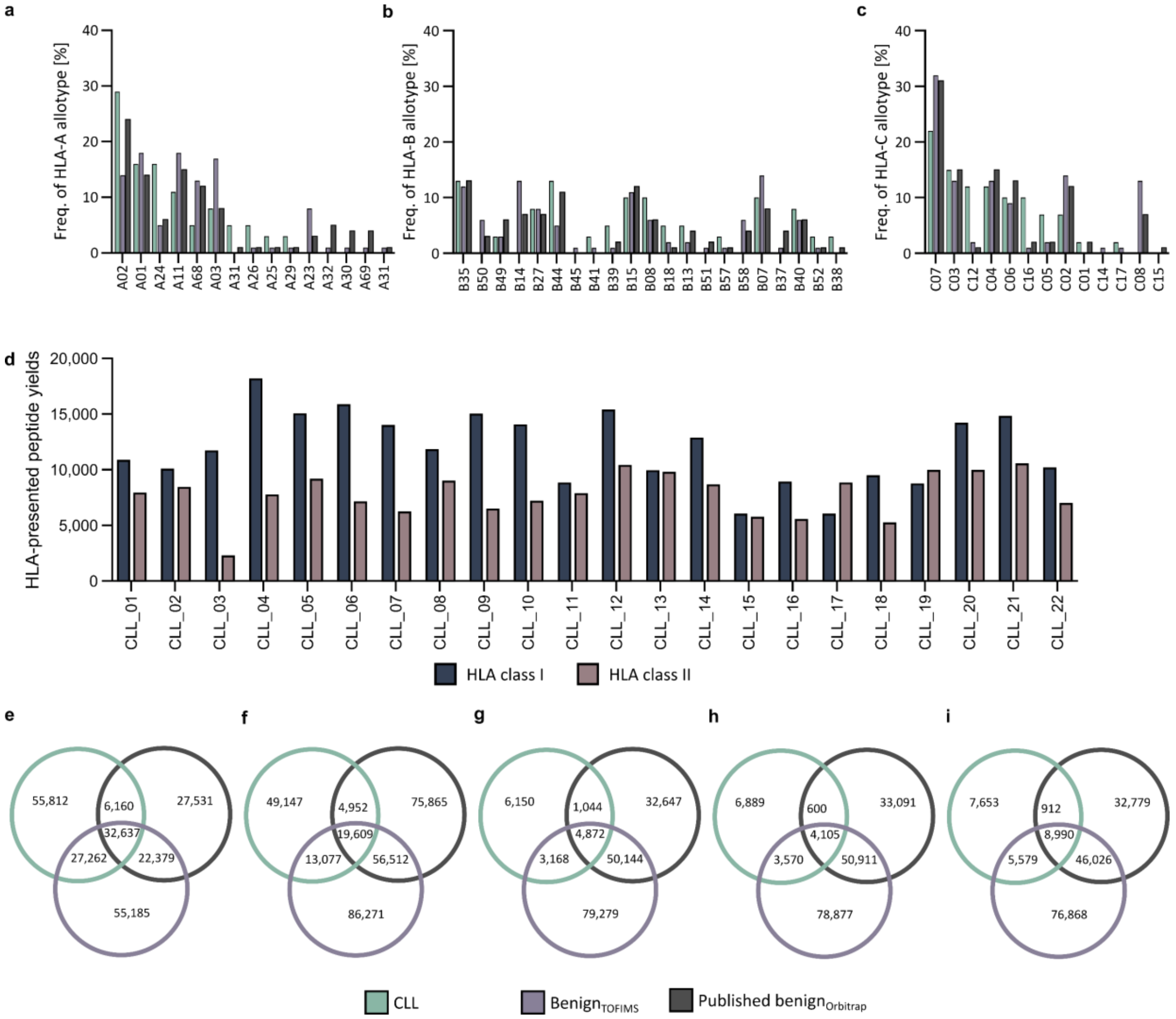

Supplementary Fig. 4 | Characterization of TOF_{IMS}-based CLL dataset. a, b, c, Comparison of HLA-A (a), HLA-B (b) and HLA-C (c) allotypes included in the CLL sample cohort with allotypes included in the $benign_{TOFIMS}$ and in published benign repositories. **d,** HLA class I and HLA class II ligand yields of the primary CLL samples (n = 22). **e, f, g, h, i,** overlap analysis of CLL, $benign_{TOFIMS}$ and published benign repositories identified HLA class I (e), HLA class II (f), HLA-A*02 (g), HLA-B*35 (h) and HLA-C*07 (i) ligands. Abbreviations: HLA, Human leukocyte antigen; CLL, chronic lymphocytic leukemia; freq, frequency. Source data are provided as a Source Data file.
