# Supplementary material for: TOFIMS mass spectrometry-based immunopeptidomics refines tumor antigen identification
Source: Nat Commun. 2023 Nov 17;14:7472. doi: 10.1038/s41467-023-42692-7 (PMC10656517; doi:10.1038/s41467-023-42692-7)
Supplement: Supplementary file 3 — Description of Additional Supplementary Files [file 41467_2023_42692_MOESM3_ESM.pdf]

File Name: Supplementary Data 1

Description: Supplementary Data 1 contains detailed description concerning method development.

File Name: Supplementary Data 2

Description: Supplementary Data 2 contains information on malignant and benign primary samples included in the benign$_{TOFIMS}$, orbitrap comparison and CLL sample cohort. Information on patient samples include age and sex after informed consent.

File Name: Supplementary Data 3

Description: Supplementary Data 3 contains information on peptide yields of all samples included in bening$_{TOFIMS}$ and CLL sample cohort. HLA class I ligand yields, HLA class II-presented peptide yields, number of peptide spectrum matches (PSM) and number of source proteins are listed for each sample.
